# Why Has the Continuous Decline in German Suicide Rates Stopped in 2007?

**DOI:** 10.1371/journal.pone.0071589

**Published:** 2013-08-14

**Authors:** Ulrich Hegerl, Roland Mergl, Gülcihan Doganay, Konrad Reschke, Christine Rummel-Kluge

**Affiliations:** 1 Department of Psychiatry and Psychotherapy, University of Leipzig, Leipzig, Germany; 2 Department of Clinical Psychology and Psychotherapy, University of Leipzig, Leipzig, Germany; University of Wuerzburg, Germany

## Abstract

**Background:**

Whereas German suicide rates had a clear decreasing tendency between 1991 and 2006, they increased from 2007 to 2010. Deeper analyses of suicide data might help to understand better this change. The aim of this study was to analyze 1) whether recent trends can be related to changes in specific suicide methods and diverge by gender and age; 2) whether the decrease of suicide rates before 2007 as well as the increase from 2007 to 2010 are driven by the same suicide method.

**Methods:**

Analyses were based on suicide data from the Federal Statistical Office of Germany. For 1998–2010, 136.583 suicide cases of men and women with known age and suicide method could be identified. These data were analyzed by joinpoint regression analysis, allowing identification of the best fitting point in time (“joinpoint”) at which the suicide rate significantly changes in magnitude or direction.

**Results:**

The national downward trend between 1998 and 2007 was mainly due to corresponding changes in self-poisoning by other means than drugs (e.g., pesticides) (annual percentage change (APC) ≤ −4.33), drowning (APC ≤ −2.73), hanging (APC ≤ −2.69) and suicides by firearms (APC ≤ −1.46) in both genders. Regarding the overall increase of age-adjusted suicide rates in Germany 2007–2010, mainly the increase of self-poisoning (e.g., by drugs) and “being overrun” (APC ≥ 1.50) contributed to this trend.

**Limitations:**

The true suicide rates might have been underestimated because of errors in the official death certificates.

**Conclusions:**

Increase in suicide rates in Germany since 2007 went along with corresponding changes for “being overrun” and “self-poisoning”. Copycat suicides following the railway suicide of the goalkeeper Robert Enke partly contributed to the results. Thus, prevention of Werther effects and limitation of the availability of high pack sizes for drugs are of special relevance for the reversal of this trend.

## Introduction

Regarding the last two decades of the 20^th^ century, there was a rather consistent decline of suicide rates in Europe, with the former states of the Soviet Union being a remarkable exception [1]. Better detection and improved therapy of mental disorders have been discussed as possible reasons for this favourable trend [2], [3]. Regarding German suicide rates from 1991 to 2002, a marked downward tendency (percentage change in men: −24%; percentage change in women: −34%) was demonstrated [4]. A more detailed analysis of the German suicide data in this period [5] revealed significant declines of the frequency of suicides for self-poisoning, drowning, hanging, strangling or suffocation whereas the frequency of other suicide methods (firearms, cutting instruments) did not significantly change. German suicide rates further decreased between 2002 and 2006; however, from 2007 to 2010 a continuous increase of the German suicide rates was recorded.

Several reasons such as the economic crisis, changes in the prevalence of depression and other psychiatric disorders or demographic changes are discussed in this context. The intense media coverage concerning the suicide of the German national football goal keeper Robert Enke on Nov. 10, 2009 is also of interest. This media coverage has triggered not only short increases of railway suicides [6], but might have also contributed to long-term increases in suicide rates. Own analyses showed that Enke’s suicide coincided with a 19% increase of railway suicidal acts in Germany in the subsequent two years, as compared to the two-year period before [7]. In line with the concept that identification with the suicide victim increases the suicide risk, the increase in railway suicides was more pronounced in men than women. The broad public discussion might even have increased the suicide rates without railway suicides by lowering the threshold and increasing the cognitive availability of the option of suicide for despaired and hopeless people. Deeper analyses of suicide data might help to better understand this change in suicide trends and sharpen suicide preventive strategies.

The aim of this study was to analyze.

1) whether recent trends can be related to changes in some suicide methods and diverge by gender and age.

2) whether the decrease of suicide rates before 2007 as well as the increase from 2007 to 2010 are driven by the same suicide method.

Both aims could be achieved.

## Methods

### Sample and Data Collection

1998 was chosen as the first year of the selected time interval (1998–2010) because the coding of suicide methods according to the ICD-10 (International Classification of Diseases, 10^th^ revision [8]) categories X60 to X84 was introduced in this year in Germany and it would have been problematic to merge data which have been differently coded.

Suicide was defined as deathly “intentional self-harm” according to the ICD-10 categories X60 to X84. Death certifications were the basis for cause of death data, as collected by the Federal Statistical Office of Germany; the authors gained these data from the German Report on Health Statistics [9]. The following information was available: age category (<1 year; 1 to 4.99 years; further 5-year intervals up to 89.99 years; 90 years and older), gender (male/female), year of the suicide (1998 to 2010), suicide method and location of the suicidal act (one of the 16 states of Germany).

### Suicide Methods

The suicide methods were combined as follows: “Self-Poisoning by psychotropic drugs” (according to ICD-10: X61-X63); “Self-Poisoning by other drugs” (according to ICD-10: X60, X64); “Self-Poisoning by other means” (according to ICD-10: X65-X69); “Hanging, strangling or suffocation” (according to ICD-10: X70); “Drowning or submersion” (according to ICD-10: X71); “Suicide by firearms” (according to ICD-10: X72-X74); “Stab with sharp instrument” (according to ICD-10: X78); “Jumping from high places” (according to ICD-10: X80); “Being overrun” (according to ICD-10: X81); “Other suicide methods” (according to ICD-10: X75-X77, X79, X82-X84).

### Age-adjustment

The age-adjusted German annual suicide rates were estimated from 1998 to 2010 by the direct method of standardization, for men and women separately, due to changes of the age structure of the German population since 1998. All suicide rates were age-standardized using the German population of the calendar year 1998 as reference population. Population data were available from the Federal Statistical Office of Germany [9]. For age standardization, we utilized the following age groups: <15 years; 15 to 19.99 years; further 5-year intervals up to 89.99 years; 90 years and older. Suicide rates were calculated per 100.000 persons per year.

### Data Analysis

For the analysis of age-adjusted suicide rate trends in Germany between 1998 and 2010, joinpoint regression analysis was selected, with the year of suicide being the independent variable. This method allows detection of these years in the study period which were associated with a significant change of the temporal trend. In this context, the joinpoint was identical with the best fitting time point at which the (age-adjusted) suicide rate significantly changed. The joinpoint regression analysis began with a zero joinpoint (a straight regression line). Next, it was tested whether one or more joinpoints had to be added to the model because they were statistically significant (with the maximum number of joinpoints being two). In a further step, the Annual Percentage Change (APC) with the corresponding 95% confidence interval (CI) was estimated for each identified trend. For this purpose, the regression line was fitted to the natural logarithm of the age-adjusted suicide rate, following the formula: ln(age-adjusted suicide rate) = α+β*x, with β representing the year of suicide. Based on this regression equation, APC was computed according to the formula: APC = (e^β^ −1) * 100. If the value ‘zero’ was not inside the 95% CI of the APC, the APC value was statistically significant.

By using this approach, we identified joinpoints for the age-adjusted German annual suicide rates from 1998 to 2010, for men and women separately. Thus, sub-periods could be defined. In a next step, we computed sub-period-specific APC with the corresponding 95% CI in order to quantify different method- and gender-specific suicide trends during the period 1998–2010. In this way, it could be investigated whether method-specific suicide trends reflected the national suicide trend or not.

In order to compare suicide trends during the period 1998–2010 in men to those in women, we also computed the age-adjusted Average Annual Percentage Change (AAPC) with the corresponding 95% CI. The AAPC can be defined as “a summary measure that is computed, over a fixed interval, as a weighted average of the slope coefficients of the joinpoint regression with the weights equal to the length of each detected segment over the interval” [10] (p. 90). For zero joinpoints, APC and AAPC are identical.

Two sets of suicide trend data (for men versus women) were compared by the test of parallelism [11]. By using this test, it can be examined whether two regression mean functions are parallel or not.

For the joinpoint regression analysis, the Joinpoint Regression Program™, Version 3. 5.2. (Statistical Research and Applications Branch of the National Cancer Institute [12]) was utilized. All further statistical tests were performed with the statistical software package for IBM SPSS Statistics 20™ for Windows (IBM, New York, USA). The significance level was set at α  = 0.05. All statistical tests were two-tailed.

## Results

### Overall Suicide Mortality Trends in Germany (1998–2010)

**Figure 1:**
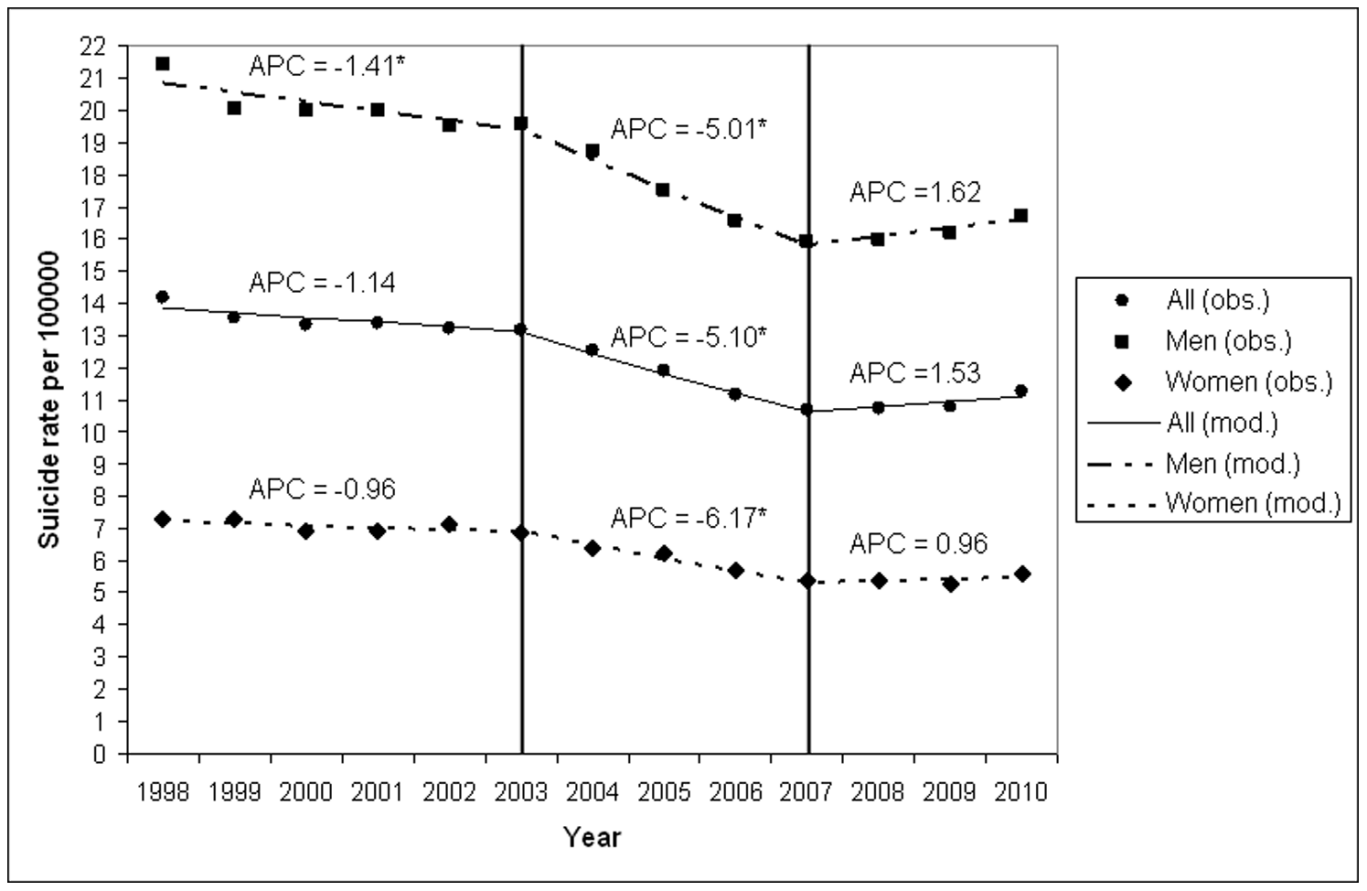
Suicide mortality in Germany (1998–2010). Based on the results of joinpoint regression analyses, age-adjusted suicide rates in all subjects (no symbol), males (squares) and females (rhombi), corresponding trends (lines) and Annual Percentage Changes (APC) are given. * p<0.05.

Regarding the time span 1998–2010 in Germany, the age-adjusted suicide rate significantly decreased until 2007 (see Figure 1). In absolute numbers this means a reduction from 11644 suicides in the year 1998 to 9402 in 2007. Interestingly, the APC for the period 2003–2007 (-5.10; 95% CI: −7.69 to −2.44; p  = 0.005) was more pronounced than the APC for the preceding period (1998–2003: −1.14; 95% CI: −2.27 to 0.00; p  = 0.05), with the change in trend in 2003 being significant (Z = -3.51; p  = 0.02). In 2007, an even more significant trend change occurred (Z  = 4.36; p  = 0.007): Since 2007, there was a continuous increase of the age-adjusted suicide rates per 100000 by 1.5% per year after a clear decline since 1991 [4] (2007–2010: APC  = 1.53; 95% CI: −1.34 to 4.49) with the slope failing to be statistically significant (p  = 0.23). In absolute numbers this corresponds to an increase of 9402 suicides in 2007 to 10021 suicides in 2010.

Similar changes in suicide trends were found for males and females (see Figure 1).

In both males and females, the AAPC for the total period (1998–2010) were negative and significantly different from 0 (males: AAPC = -1.89; 95% CI: −3.00 to −0.76; females: AAPC = -2.26; 95% CI: −3.87 to −0.62). The decline in suicide rates did not differ between males and females (AAPC difference between male and female:-0.37; 95% CI: −2.40 to 1.64; p  = 0.71). In line with this finding, the joinpoint regression mean functions in male and female suicides were comparable, as revealed by the test for parallelism (p  = 0.19).

### Differences between Suicide Methods in Suicide Mortality Trends

**Figure 2:**
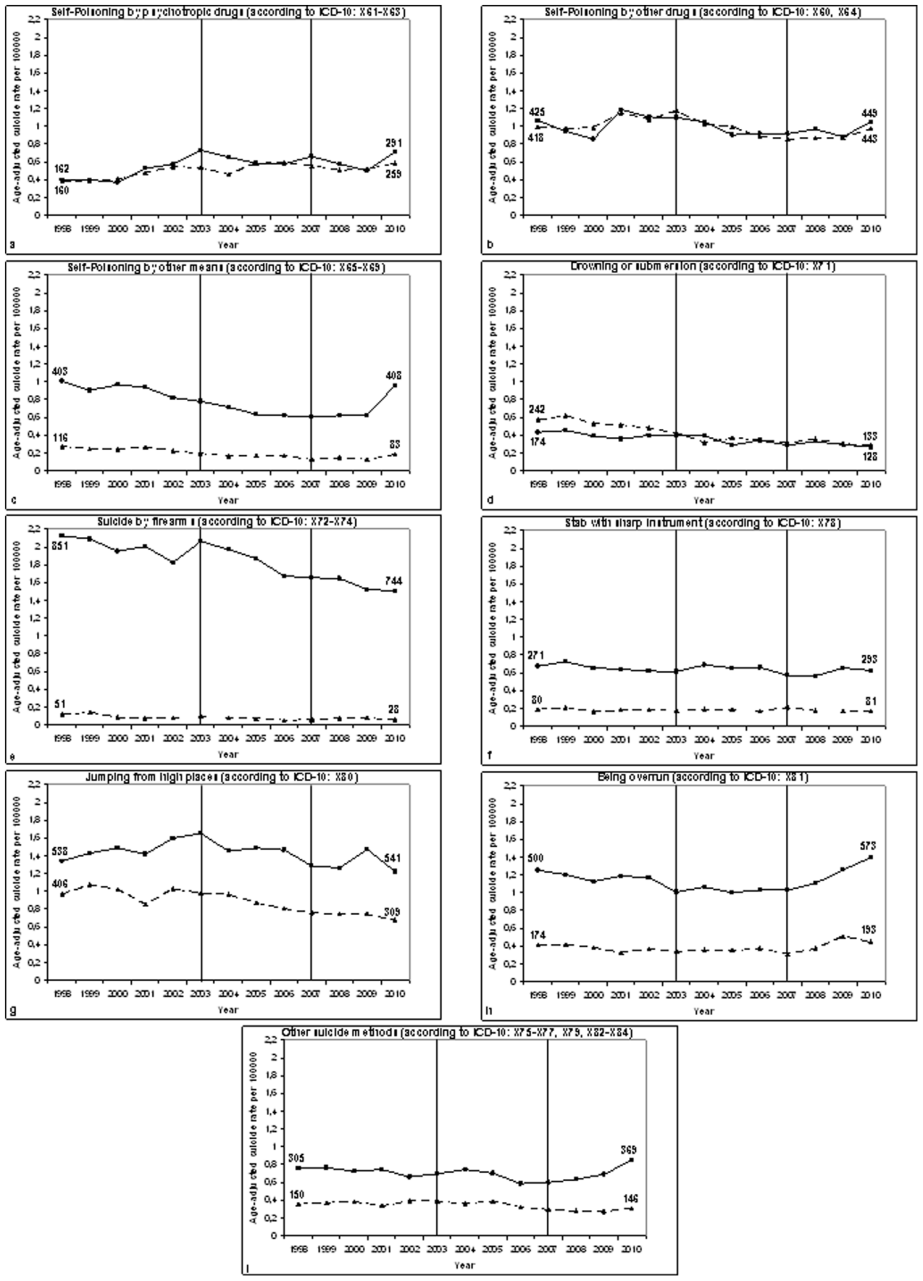
Age-adjusted suicide rates (per 100000 inhabitants) in Germany (1998–2010) for males and females. The rates (reference year: 1998) were stratified for several suicide methods: **a)** Self-Poisoning by psychotropic drugs (according to ICD-10: X61-X63); **b)** Self-Poisoning by other drugs (according to ICD-10: X60, X64); **c)** Self-Poisoning by other means (according to ICD-10: X65-X69); **d)** Drowning or submersion (according to ICD-10: X71); **e)** Suicides by firearms (according to ICD-10: X72-X74); **f)** Stab with sharp instrument (according to ICD-10: X78); **g)** Jumping from high places (according to ICD-10: X80); **h)** Being overrun (according to ICD-10: X81); **i)** Other suicide methods (according to ICD-10: X75-X77, X79, X82-X84). –– Age-adjusted suicide rates for men (with squares); - - - Age-adjusted suicide rates for women (with triangles). Vertical lines represent the joinpoints which had been identified for the overall suicide mortality trends in Germany 1998–2010. The numbers in the figures represent suicide frequencies in males and females, for the years 1998 and 2010, respectively.

Can the increase of suicides in Germany between 2007 and 2010 be related to changes of specific suicide methods and does it differ by gender and age? The unfavourable change of the suicide trend in Germany starting in 2007 (from decrease to moderate, but non-significant increase) coincided with increases of suicides by “being overrun”, “self-poisoning by psychotropic drugs”, “self-poisoning by other drugs”, “self-poisoning by other means” and “other suicide methods” in both men and women (see Table 1 and Figures 2a–c,h,i).

**Table 1:** Annual percentage change in age-adjusted mortality rates for different suicide methods (+95% confidence intervals) (1998–2010) in Germany.

Suicide methods	1998–2003			2003–2007			2007–2010		
	Men	Women	Difference	Men	Women	Difference	Men	Women	Difference
**Poisoning by** **psychotropic drugs**	**14.2** (5.9; 23.1)(p = 0.008)	**8.6** (4.7; 12.6)(p = 0.003)	5.6 (−0.9; 10.9)(p = 0.10)	−3.1 (−11.3; 5.9)(p = 0.34)	3.2 (−6.1; 13.4)(p = 0.37)	−6.3 (−14.3; 1.7)(p = 0.12)	1.6 (−27.4; 42.3)(p = 0.86)	1.5 (−10.8; 15.5)(p = 0.67)	0.1 (−16.3; 16.6)(p = 0.99)
**Poisoning by** **other drugs**	2.7 (−4.9; 10.8)(p = 0.40)	**3.7** (0.3; 7.3)(p = 0.04)	−1.1 (−7.0; 4.9)(p = 0.73)	−**4.9** (−9.5; −0.1)(p = 0.05)	−**7.5** (−10.5; −4.5)(p = 0.004)	2.6 (−0.8; 6.4)(p = 0.13)	3.4 (−10.0; 18.8)(p = 0.41)	3.9 (−2.4; 10.7)(p = 0.12)	−0.5 (−7.5; 6.4)(p = 0.88)
**Poisoning by** **other means**	−**4.3** (−7.9; −0.7)(p = 0.03)	−4.5 (−9.5; 0.8)(p = 0.08)	0.2 (−4.5; 4.8)(p = 0.94)	−**6.2** (−9.7; −2.6)(p = 0.01)	−7.2 (−15.6; 2.2)(p = 0.09)	0.9 (−5.4; 7.3)(p = 0.76)	16.4 (−13.6; 56.7)(p = 0.16)	9.3 (−18.1; 45.7)(p = 0.32)	7.1 (−12.6; 25.2)(p = 0.51)
**Hanging**	−**2.8** (−4.7; −0.8)(p = 0.02)	−**2.7** (−4.5; −0.9)(p = 0.02)	−0.1 (−2.0; 1.8)(p = 0.92)	−**6.0** (−6.8; −5.2)(p = 0.0002)	−**8.2** (−10.6; −5.8)(p = 0.002)	**2.2** (0.7; 4.1)(p = 0.01)	−0.5 (−2.2; 1.2)(p = 0.32)	0.3 (−13.3; 15.9)(p = 0.95)	−0.8 (−7.4; 5.9)(p = 0.82)
**Drowning**	−2.7 (−7.5; 2.2)(p = 0.20)	−**6.2** (−9.8; −2.4)(p = 0.01)	3.5 (−0.8; 8.1)(p = 0.11)	−7.2 (−16.7; 3.4)(p = 0.12)	−5.6 (−14.9; 4.8)(p = 0.18)	−1.6 (−11.0; 7.5)(p = 0.71)	−3.1 (−17.7; 4.3)(p = 0.50)	−4.3 (−20.9; 15.8)(p = 0.42)	1.3 (−10.2; 12.8)(p = 0.82)
**Suicide by** **firearms**	−1.5 (−4.8; 2.0)(p = 0.30)	−7.6 (−18.7; 5.0)(p = 0.16)	6.1 (−2.9; 15.7)(p = 0.18)	−**5.8** (−8.0; −3.7)(p = 0.004)	−**12.5** (−19.3; −5.2)(p = 0.01)	**6.7** (2.2; 12.5)(p = 0.005)	−3.6 (−7.7; 0.7)(p = 0.07)	1.7 (−27.5; 42.5)(p = 0.85)	−5.3 (−20.8; 10.2)(p = 0.50)
**Stab**	−**2.8** (−5.4; −0.2)(p = 0.04)	−1.9 (−7.6; 4.1)(p = 0.42)	−0.9 (−5.6; 3.7)(p = 0.69)	−1.8 (−9.4; 6.4)(p = 0.52)	2.3 (−5.4; 10.6)(p = 0.43)	−4.1 (−11.0; 2.8)(p = 0.25)	4.2 (−6.6; 16.4)(p = 0.25)	−5.8 (−15.2; 4.5)(p = 0.13)	**10.1** (3.2; 17.1)(p = 0.004)
**Jumping**	**3.9** (1.4; 6.5)(p = 0.01)	−0.6 (−6.0; 5.1)(p = 0.76)	**4.5** (0.2; 8.8)(p = 0.04)	−4.8 (−9.4; 0.0)(p = 0.0501)	−**6.7** (−9.1; −4.2)(p = 0.003)	1.9 (−1.4; 5.5)(p = 0.25)	0.2 (−17.8; 22.1)(p = 0.97)	−3.2 (−9.0; 2.9)(p = 0.15)	3.4 (−6.0; 12.9)(p = 0.48)
**Being overrun**	−2.9 (−6.2; 0.4)(p = 0.07)	−**4.0** (−7.9; −0.01)(p = 0.05)	1.1 (−2.7; 4.9)(p = 0.56)	0.04 (−2.7; 2.9)(p = 0.96)	−0.5 (−7.5; 6.9)(p = 0.83)	0.6 (−4.2; 5.4)(p = 0.81)	**11.0** (7.4; 14.8)(p = 0.005)	13.7 (−15.2; 52.6)(p = 0.20)	−2.7 (−15.9; 11.1)(p = 0.73)
**Other suicide** **methods**	−**2.3** (−4.6; −0.01)(p = 0.05)	1.5 (−2.3; 5.4)(p = 0.34)	−**3.8** (−6.9; −0.7)(p = 0.02)	−5.3 (−11.8; 1.8)(p = 0.10)	−6.4 (−13.6; 1.4)(p = 0.08)	−1.1 (−5.4; 7.8)(p = 0.72)	**12.4** (0.5; 25.8)(p = 0.05)	1.3 (−9.0; 12.8)(p = 0.65)	**11.1** (3.3; 17.5)(p = 0.004)

**Notes:** Significant findings are in bold.

The increase of age-adjusted suicide rates for “being overrun” (Figure 2h) between 2007 and 2010 was very pronounced in both sexes (APC ≥ 11.04), but only significant for men. The increase was 11.04 in men and 13.73 in women, the difference (−2.69) being not significant. In absolute numbers, suicides by being overrun increased from 572 in 2007 to 766 in 2010 (men: 431 to 573, women: 141 to 193).

For “self-poisoning by psychotropic drugs” (Figure 2a), the age-adjusted suicide rate increased in both men and women between 2007 and 2010 (men: APC  = 1.61; p  = 0.86; women: APC  = 1.50; p  = 0.67). Age group specific analysis revealed that this trend was most pronounced in younger females (being younger than 26 years) (APC  = 41.30; p  = 0.08) (see Table S1).

The age-adjusted suicide rates for “poisoning by other drugs” (Figure 2b) were characterized by a marked increase between 2007 and 2010 (APC ≥ 3.41) in both men and women. This trend was age-dependent; however, the age group specific trends failed to be significant. For this reason, they are only presented in the appendix (see Table S1).

An even stronger increase of the age-adjusted suicide rates between 2007 and 2010 was found for “self-poisoning by other means” in men (APC  = 16.37; p  = 0.16; see also Figure 2c): The annual percentage increase of this method since 2007, albeit not being significant, is clearly higher than the corresponding value for all suicide methods in men (1.62). An analogous trend was registered for women (APC  = 9.25; p  = 0.32). According to age group analyses, this increase was strongest for young subjects (≤ 25 years) using “self-poisoning by other means” as suicide method (men: APC  = 60.21; p  = 0.03; women: APC  = 66.90; p  = 0.20) (see Table S1).

More detailed analysis demonstrated that the increase of suicides from 2007 to 2010 for “self-poisoning by other means” was especially pronounced for “intentional self-poisoning by and exposure to other gases and vapours than organic solvents and halogenated hydrocarbons and their vapours” (men: APC  = 22.74; p  = 0.09; women: APC  = 36.25; p  = 0.05).

Regarding “other suicide methods”, increase of the corresponding suicide rates between 2007 and 2010 was present in men and women, but only significant in men (APC  = 12.44; p  = 0.05; see also Figure 2i). This finding can be explained by significant increases of suicides by this method in older men (45–64 years: APC  = 12.48; p  = 0.05; 65 years and older: APC  = 4.18; p  = 0.02) (see Table S1).

In order to specify this effect, additional analyses were performed for the ICD-10 categories “X76” (“Intentional self-harm by smoke, fire and flames“) and “X82” (“Intentional self-harm by crashing of motor vehicle“) in men (for analysis of the categories “X75”, “X77” and “X79” the numbers (<8 per year) were too low whereas the residual categories “X83” and “X84” could not be well interpreted). In both cases, marked, but non-significant increases of the age-adjusted suicide rates were present (ICD-10 X76: APC  = 8.93 (2007∶40 registered suicides; 2010∶53 registered suicides); p  = 0.25; ICD-10 X82: APC  = 8.70 (2007∶47 registered suicides; 2010∶58 registered suicides); p  = 0.07).

**Figure 3:**
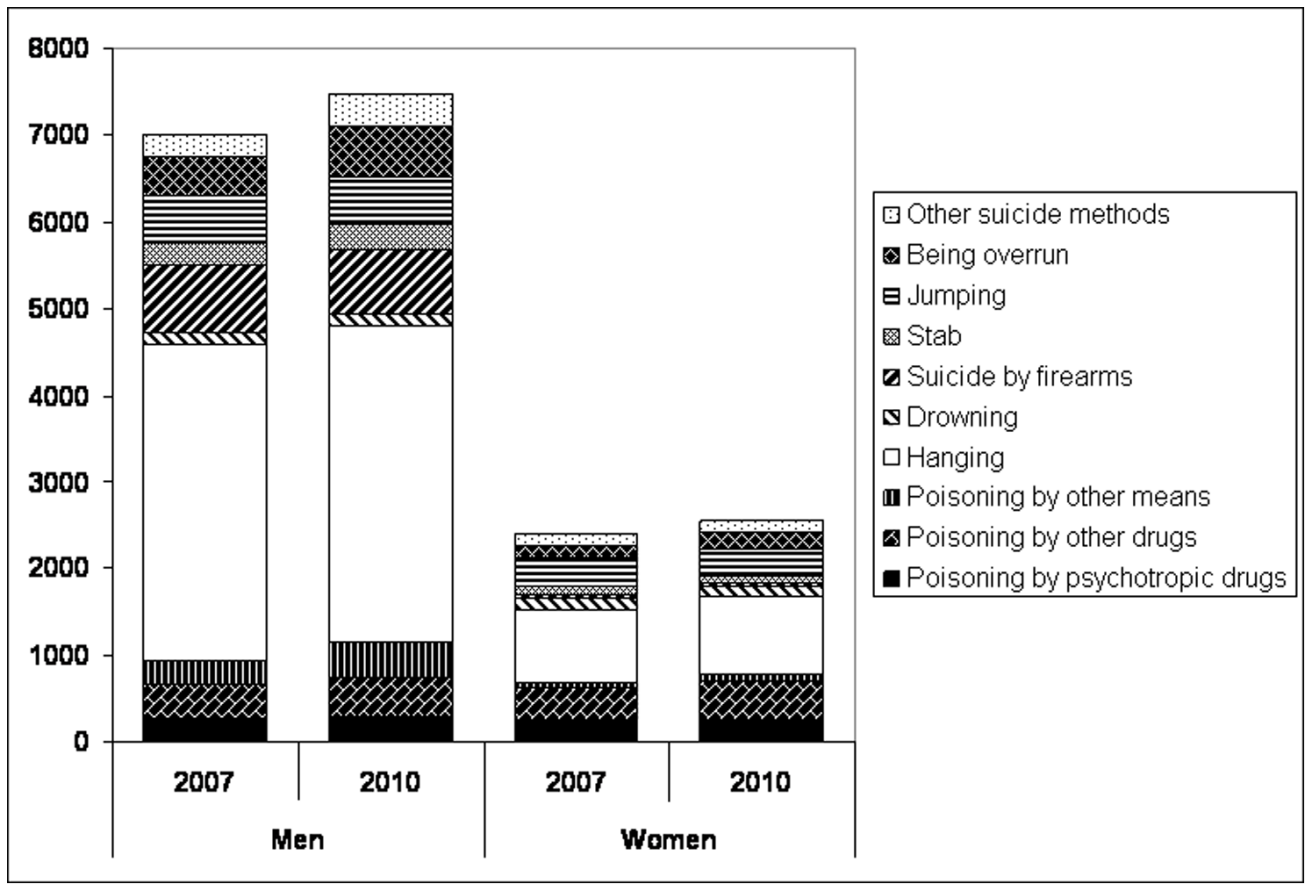
Changes of method-specific suicide frequencies between 2007 and 2010 in men and women in Germany.

The changes of the method-specific suicide frequencies between 2007 and 2010 in both men and women in Germany are visualized by Figure 3.

For “being overrun” as suicide method, a very pronounced increase of the absolute frequencies between 2007 and 2010 was apparent (men: +32.9%; women: +36.9%). The relative proportion of this method regarding suicides in Germany only moderately increased in this period (men: 6.15-7.67%; women: 5.89–7.55%). This increase already started before Enke’s suicide. Additional analyses revealed that the most pronounced increase within this period was present between 2008 and 2009 (men: 13.64%; women: 37.84%), with the corresponding increase between 2007 and 2008 being much lower (men: 6.80%; women: 15.63%).

#### Is the decrease of suicide rates before 2007 as well as the increase from 2007 to 2010 driven by the same suicide method?

**Figure 4:**
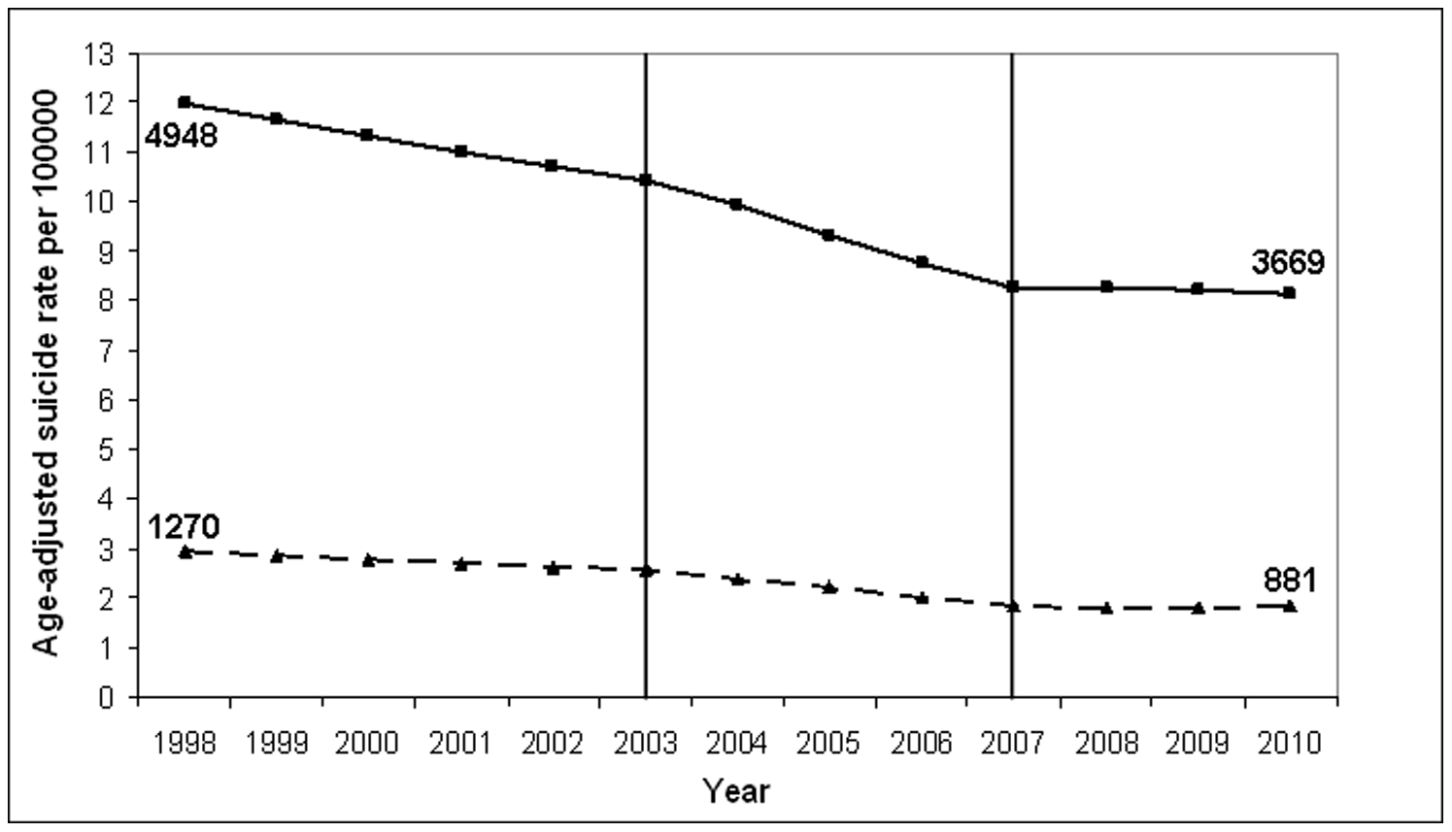
Age-adjusted suicide rates for hanging in Germany (1998–2010) for males and females, separately. Suicide rate per 100000 inhabitants (reference year: 1998); suicide method: “Hanging, strangling or suffocation” (according to ICD-10: X70). –– Age-adjusted suicide rates for men (with squares); …….Age-adjusted suicide rates for women (with triangles). Vertical lines represent the joinpoints which had been identified for the overall suicide mortality trends in Germany 1998–2010. The numbers in the figure represent suicide frequencies in males and females, for the years 1998 and 2010, respectively. obs. = observed values; mod. = modelled values (according to the joinpoint regression analysis).

The national downward trend of suicides in Germany between 1998 and 2007 was mainly due to corresponding changes for self-poisoning by other means (annual percentage change (APC) ≤ −4.33), drowning (APC ≤ −2.73), hanging (APC ≤ −2.69) (see Figure 4) and suicides by firearms (APC ≤ −1.46) in both genders. The same is true for suicides with sharp instruments in the subgroup of men (APC ≤ −1.81), followed by an increase in the period 2007–2010 (APC = 4.24; p  = 0.25). The gender difference in suicide mortality trends for stabs with sharp objects (2007–2010) was significant (increase in men, decrease in women) (APC difference  = 10.06; p  = 0.004). Nevertheless, it must be emphasized that neither the percentage change in men nor that in women was significant.

Regarding the suicide trend in men in Germany (2003–2007: pronounced significant decline; 2007–2010: moderate, but non-significant increase), it was associated with similar changes of the suicide method “self-poisoning by other means” in men (2003–2007: APC = −6.23; p  = 0.01; 2007–2010: APC  = 16.37; p  = 0.16; see also Figure 2c). The same is true for other suicide methods in men (2003–2007: APC = −5.25; p  = 0.10; 2007–2010: APC  = 12.44; p  = 0.05; see also Figure 2i).

For both men and women, the suicide methods “self-poisoning by other means” and “suicide by firearms” reflect the suicide trend for the period 2003–2010 in Germany (see Figure 2c and e) (2003–2007: pronounced decline; 2007–2010: moderate increase).

As self-poisoning and being overrun were associated with increased suicide rates in the period 2007–2010 in Germany in both males and females (see above), the corresponding changes between 2003 and 2007 are of special interest:

Self-poisoning by psychotropic drugs (Figure 2a) decreased in men between 2003 and 2007 (APC = −3.10; p  = 0.34) whereas positive, but non-significant APC values were found for women in the same period (APC  = 3.19; p  = 0.37).

The age-adjusted suicide rates for “self-poisoning by other drugs” (Figure 2b) were characterized by a pronounced decline between 2003 and 2007 (APC ≤ −4.90) in both men and women.

Being overrun (Figure 2h) was found to be a suicide method which was nearly constant between 2003 and 2007 (men: APC  = 0.04; p  = 0.96; women: APC = −0.54; p  = 0.83).

## Discussion

### Overall Suicide Mortality Trends in Germany (1998–2010)

Trend analysis revealed that between 1998 and 2010 three different periods can be separated: a slower decline between 1998 and 2003, a stronger decline in the following period from 2003 to 2007 and an abrupt change in trend with slow increase in suicide rates between 2007 and 2010. The trend change in 2007 was more pronounced than that in 2003. These different trends were found both for males and females.

It can be assumed that there are several factors contributing to these changes in suicide rates as well as changes in trends. It is of interest that the decline in suicide rates from 24.0/100000 in the early eighties to 12.3/100000 in 2010 (data source: Federal Statistical Office of Germany) was associated with a clear increase in sick leaves or early retirement because of depression and other mental disorders according to data from the Federal German Health report [13]. Also the prescription rates of antidepressants strongly increased during these three decades. These changes do not appear to reflect an increase in the prevalence of depression or other psychiatric disorders, but the desirable development that more and more depressed patients seek help, that depression is better diagnosed and treated by health professionals and that depression is more often correctly identified as depression and not hidden behind less stigmatized terms such as burnout, fibromyalgia, chronic lower back pain or other somatic diagnoses. Indeed, population based epidemiological studies do not provide evidence for an increase in depressive disorders [14], [15].

One factor explaining the steeper decline in suicide rates from 2003–2007 compared to 1998–2002 could be that many community based multifaceted interventions targeting depression and suicidality and public campaigns have been started by the German Alliance against Depression [16] since 2002 stimulated by the promising results of the pilot project “Nuremberg Alliance against Depression” [17]. It will be of interest to compare suicide trends in regions with versus without such regional interventions in order to analyze the possible impact of this factor on suicide trends.

Regarding the subsequent remarkable change in suicide trends with increase of suicide rates (2007–2010), the current economic crisis in 2008 is sometimes discussed as a possible factor. However, this appears to be unlikely because increasing suicide rates coincided with a decline of the unemployment rates in Germany in the period 2007–2010 [7].

An increase of major depression and other suicide-related mental disorders is also unlikely to explain this change in trend. The increases in prevalence of mental disorders in the statistics of health insurances or social systems were also observable during the decades with decreasing suicide rates and, as discussed above, are indicating better care of depressed patients and not necessarily increases in prevalence of depression in the general population [14], [15].

The trend analyses in subgroups stratified by gender and suicide methods provided further insights in the underlying processes.

### Can the Increase of Suicides in Germany between 2007 and 2010 be Related to Specific Suicide Methods, Age Groups and Gender Aspects?

The overall increase of age-adjusted suicide rates in Germany in 2007–2010 coincided with the following suicide methods in both men and women: self-poisoning (independently from the means); being overrun and the residual category of “other suicide methods”. This increase was very pronounced for being overrun, with the annual percentage change being significant in males (APC = 11.04; p = 0.005). Especially men aged 25–64 were characterized by a strong increase of suicides by being overrun between 2007 and 2010 (APC ≥ 11.95; p ≤ 0.05). Similar findings were found for women aged 25–64 years, although failing to be statistically significant. This result is in line with previous findings suggesting that the railway suicide by the goalkeeper of the German football team Robert Enke in November 2009 was not only associated with a short-term increase of railway suicides in Germany within 28 days, but also with long-term (two-year) effects [7]. The long-term increase of railway suicidal acts after Enke’s death was present in both men and women, but more pronounced in men [7]. Although the German data for being overrun as suicide method not only comprise railway suicides, but also suicides with other means of transport being involved (cars, tramway and subway), the pronounced Werther effect following Enke’s suicide can be considered to be a part of the increasing trend regarding the age-adjusted suicide rates for being overrun in Germany in the period 2007–2010. Indeed, the most pronounced increase within this period was found between 2008 and 2009 (men: 13.64%; women: 37.84%), with the corresponding increase between 2007 and 2008 being much lower (men: 6.80%; women: 15.63%). In this context, it is interesting that the increase of the absolute frequencies for suicides by being overrun between 2007 and 2010 was found to be very pronounced (over 30%), whereas the increase of the percentage of this suicide method regarding all suicides per year was only moderate in this period (men: 1.5% (increase of 6.2 to 7.7%); women: 1.7% (increase of 5.9 to 7.6%)). Thus, it is unlikely that the increase of railway and related suicides between 2007 and 2010 merely reflects a change in the choice of suicide methods. Instead, it is more probable that increased frequencies of being overrun following Enke’s suicide contributed to the overall increase of suicide rates in Germany in this period. This is in line with findings suggesting that the Werther effect reflects a true increase of suicide rates after the suicide of a prominent person (for review see [18]). The improvement of mass media reports about celebrity suicides like that committed by Robert Enke represents an important approach to prevent the Werther effect [19].

Regarding self-poisoning, the increase of these suicide methods since 2007 in both men and women in Germany underlines the necessity to intensify suicide preventive activities in this field. One option would be to limit the availability of toxic substances, e.g. by lower pack sizes for drugs, as earlier proposed [5].

Because of low numbers, the gender-specific trends for suicides by firearms (decrease in men (2007∶778 cases; 2010∶744 cases), slight increase in women (2007∶27 cases; 2010∶28 cases)) were not significant and cannot be interpreted.

### Is the Decrease of Suicide Rates before 2007 as Well as the Increase from 2007 to 2010 Driven by the Same Suicide Method?

Only few suicide methods were found to drive both the decrease in suicide rates in the years before 2007 and the increase of suicides from 2007 to 2010 in Germany: for men “self-poisoning by other means than drugs”, especially “intentional self-poisoning by and exposure to other gases and vapours than organic solvents and halogenated hydrocarbons and their vapours” as well as the residual category of “other suicide methods” (especially “intentional self-harm by smoke, fire and flames” and “intentional self-harm by crashing of motor vehicle”); for women “self-poisoning by other means than drugs” (especially “intentional self-poisoning by and exposure to other gases and vapours than organic solvents and halogenated hydrocarbons and their vapours”) and “suicides by firearms”.

Interestingly, the suicide trends for “being overrun” decreased between 1998 and 2003 and hardly changed in the period 2003–2007 in both men and women. Thus, individuals who chose this suicide method in Germany in 2003–2007 seem to represent a special subgroup of suicide committers which does not seem to have been touched by the global national suicide mortality trend in these years.

### Limitations

When interpreting our findings, some limitations have to be considered:

First, the analyzed time frame is rather short (1998–2010), due to the issue that suicide methods are differently defined in ICD-9 and ICD-10. Thus, we decided to analyze only years in which the ICD-10 classification of suicide methods (X60–X84) was obligatory in Germany.

Second, due to German data protection laws, other suicide risk factors than gender and age could not be analyzed on a national level.

Third, we gained our data from the German Report on Health Statistics [9]. The corresponding mortality databank is based on the official death certificates in Germany; thus, underestimation of the true number of suicides is a serious issue. However, a previous study [4] has demonstrated that the average annual percentage changes did not considerably differ for age-standardized suicide rates and the sum of suicide rates and rates for undetermined deaths in Germany, at least for individuals with an age of 25–74 years. Thus, it is not very likely that the suicide trends as reported in this article were affected by undetermined deaths in a substantial amount.

### Conclusions

Three time periods differing in trends of suicide rates can be separated in Germany: a moderate decrease of suicide rates between 1998 and 2003, a stronger decrease between 2003 and 2007, and an abrupt change to a mild increase between 2007 and 2010. It is not unlikely that better help seeking behavior of people with depression and reduction of the large diagnostic and therapeutic deficits in this area of medicine have contributed to the decline of suicide rates during the first two periods (1998–2006). The reasons why the decline was steeper from 2003–2007 compared to 1998–2003 remain unclear, although the start of community interventions and public campaigns in many regions in Germany can be discussed in this context. The national downward trend between 1998 and 2007 was mainly due to corresponding changes for self-poisoning by other means than drugs (like gases and vapours), hanging, drowning and suicides by firearms in both genders.

The abrupt change in trend with increase of age-adjusted suicide rates in Germany from 2007–2010 is remarkable. This increase was observed in spite of the fact that the statistics of health insurances and social systems indicate further increases in the number of persons with a diagnosed and treated depression or other mental disorder. The increase was mainly associated with the suicide method “being overrun”. Copycat suicides following Robert Enke’s railway suicide in November 2009 are likely to have contributed to this change in trend.

The prevention of Werther effects is therefore of high importance. To avoid unfavorable mass media reporting after celebrity suicides by early interventions should be an important element in suicide prevention programs.

## Supporting Information

Table S1 **Age-group specific changes in age-adjusted mortality rates for four suicide methods (annual percentage change > = 1) in males and females between 2007 and 2010 in Germany.**
(DOC)
