# Supplementary material for: Why Has the Continuous Decline in German Suicide Rates Stopped in 2007?
Source: PLoS One. 2013 Aug 14;8(8):e71589. doi: 10.1371/journal.pone.0071589 (PMC3743810; doi:10.1371/journal.pone.0071589)
Supplement: Table S1 — Age-group specific changes in age-adjusted mortality rates for four suicide methods (annual percentage change > = 1) in males and females between 2007 and 2010 in Germany. (DOC) [file pone.0071589.s001.doc]

**Supplementary Table 1: Age-group specific changes in age-adjusted mortality rates for four suicide methods (annual percentage change >= 1) in males and females between 2007 and 2010 in Germany**

| Suicide method | Age group 1 (<= 25 years) | | | Age group 2 (25-44 years) | | | Age group 3 (45-64 years) | | | Age group 4 (>= 65 years) | | |
| --- | --- | --- | --- | --- | --- | --- | --- | --- | --- | --- | --- | --- |
|  | **Men APC (95%CI) (p)** | **Women APC (95%CI) (p)** | **APC difference (95%CI) (p)** | **Men APC (95%CI) (p)** | **Women APC (95%CI) (p)** | **APC difference (95%CI) (p)** | **Men APC (95%CI) (p)** | **Women APC (95%CI) (p)** | **APC difference (95%CI) (p)** | **Men APC (95%CI) (p)** | **Women APC (95%CI) (p)** | **APC difference (95%CI) (p)** |
| **Poisoning by psychotropic drugs** | -2.09 (-34.17; 45.63) (p=0.84) | 41.30 (-8.44; 118.05) (p=0.08) | **-43.39**  (-63.47; -9.89) **(p=0.007)** | 1.71 (-35.01; 59.18) (p=0.89) | -4.98 (-26.72; 23.22) (p=0.49) | 6.69 (-16.78; 30.39) (p=0.57) | -0.69 (-20.32; 23.79) (p=0.91) | 2.93 (-10.49; 18.38) (p=0.47) | -3.62 (-15.46; 8.30) (p=0.55) | 8.01 (-22.32; 50.20) (p=0.42) | 0.84 (-21.62; 29.74) (p=0.90) | 7.17 (-12.03; 25.77) (p=0.48) |
| **Poisoning by other drugs** | 2.17 (-49.21; 105.52) (p=0.91) | -9.68 (-50.15; 63.64) (p=0.54) | 11.85 (-29.47; 54.12) (p=0.56) | 2.55 (-18.73; 29.41) (p=0.69) | 1.30 (-8.45; 12.09) (p=0.64) | 1.25 (-10.33; 12.79) (p=0.83) | 6.55 (-5.37; 19.96) (p=0.15) | 3.89 (-0.56; 8.55) (p=0.06) | 2.65 (-3.24; 8.28) (p=0.39) | -2.16 (-14.69; 12.23) (p=0.56) | -7.47 (-16.05; 37.57) (p=0.34) | -9.62 (-22.25; 3.49) (p=0.15) |
| **Suicide method** | **Age group 1 (<= 25 years)** | | | **Age group 2 (25-44 years)** | | | **Age group 3 (45-64 years)** | | | **Age group 4 (>= 65 years)** | | |
|  | **Men** **APC (95%CI) (p)** | **Women APC (95%CI) (p)** | **APC difference (95%CI) (p)** | **Men APC (95%CI) (p)** | **Women APC (95%CI) (p)** | **APC difference (95%CI) (p)** | **Men APC (95%CI) (p)** | **Women APC (95%CI) (p)** | **APC difference (95%CI) (p)** | **Men APC (95%CI) (p)** | **Women APC (95%CI) (p)** | **APC difference (95%CI) (p)** |
| **Poisoning by other means** | **60.21** (8.68; 136.18) **(p=0.03)** | 66.90 (-48.81; 444.21) (p=0.20) | -6.69 (-60.76; 52.58) (p=0.89) | 18.38 (-15.63; 66.09) (p=0.17) | 22.68 (-6.77; 61.42) (p=0.09) | -4.30 (-23.43; 16.29) (p=0.72) | 10.09 (-19.24; 50.06) (p=0.31) | -1.34 (-26.34; 32.14) (p=0.86) | 11.43 (-8.44; 30.36) (p=0.27) | 9.80 (-10.63; 34.89) (p=0.19) | 3.90 (-28.80; 51.60) (p=0.71) | 5.90 (-14.08; 25.12) (p=0.58) |
| **Being overrun** | 8.61 (-8.80; 29.35) (p=0.18) | 5.18 (-11.65; 25.22) (p=0.34) | 3.43 (-8.04; 14.45) (p=0.58) | 11.95 (-0.10; 25.46) (p=0.051) | 18.10 (-0.31; 39.92) (p=0.052) | -6.15 (-14.65; 3.95) (p=0.26) | **13.15** (4.56; 22.44) **(p=0.02)** | 15.14 (-22.31; 70.66) (p=0.26) | -2.00 (-20.03; 16.53) (p=0.85) | 7.23 (-11.98; 30.63) (p=0.27) | 11.03 (-34.98; 89.59) (p=0.49) | -3.80 (-29.46; 22.50) (p=0.79) |
| **Other suicide methods** | 3.87 (-8.02; 17.30) | 16.43 (-68.87; 335.38) | -12.56 (-71.75; 48.93) | 21.24 (-14.24; 71.40) | -3.01 (-32.86; 40.10) | 24.26 (-0.69; 45.33) | **12.48** (0.22; 26.24) | 4.55 (-13.78; 26.79) | 7.93 (-2.92; 17.55) | **4.18** (1.83; 6.58) | -2.11 (-33.43; 43.97) | 6.29 (-11.37; 23.82) |
| **Suicide method** | **Age group 1 (<= 25 years)** | | | **Age group 2 (25-44 years)** | | | **Age group 3 (45-64 years)** | | | **Age group 4 (>= 65 years)** | | |
|  | **Men** **APC**  **(p)** | **Women APC**  **(p)** | **APC difference (p)** | **Men APC**  **(p)** | **Women APC**  **(p)** | **APC difference (p)** | **Men APC**  **(p)** | **Women APC**  **(p)** | **APC difference (p)** | **Men APC**  **(p)** | **Women APC**  **(p)** | **APC difference (p)** |
| **Other suicide methods** | (p=0.31) | (p=0.67) | (p=0.71) | (p=0.14) | (p=0.75) | (p=0.06) | **(p=0.048)** | (p=0.43) | (p=0.16) | **(p=0.02)** | (p=0.83) | (p=0.49) |

**Notes:** APC = annual percentage change; CI = confidence interval. Significant findings are in bold.
